# Experimental Investigations into the Action of the Bromide of Potassium

**Published:** 1866-01

**Authors:** 


**Experimental Investigations into the Action of the Bromide of Potassium.**

This is the title of a very interesting paper by Dr. Roberts Bartholow, in the November number of the *Cincinnati Lancet and Observer.* The author's investigations were conducted in three directions: 1st, the chemical properties; 2d, the physiological effects; and 3d, the therapeutical uses of the salt.

The physiological effects of the article when taken into the stomach, Dr. B. sums up as follows:

"1. It proves irritant in large doses to the mucous membrane of the stomach.

"2. It is rapidly absorbed into the blood, and may be detected soon after in the urine.

"3. It acts upon the nervous centers, producing sedation, sleep, reduces the action of the heart and arteries, lowers the temperature, and diminishes the retrograde metamorphosis of tissue."

The prolonged administration of the bromide of potassium produces according to Dr. B. the following effects:

"1st. It diminishes and ultimately entirely neutralizes the sexual appetite.

"2d. It produces weakness of the muscular system.

"3d. It is irritant to the stomach if given in considerable doses, and

"4th. It interferes with the secondary assimilation, lessening the retrograde metamorphosis of tissue."

In regard to its therapeutical uses Dr. B. extols it as a *disinfectant* and *deodorizer*, as an *escharotic* in sloughing and gangrenous ulcer, phagedenic chancres, hospital gangrene, epithelioma, etc.

"The actions of the bromide of potassium physiologically considered," Dr. B. states, "consist in a sedative or contra-stimulant effect upon the nervous centers, producing as secondary phenomena, sedation of the heart, anæmia of the brain, anaphrodisiac effects and diminution of the retrograde metamorphosis of tissue. It has come into use in various functional and organic nervous disorders, and in certain sexual diseases, where a calmative and sedative influence is desired."

This article Dr. B. considers to be indicated as a hypnotic in states of nervous excitement without congestion of the nervous centers; in hysterical insomnia; in delirium tremens; in the insomnia of excitable business men, or, in general terms, in those forms of insomnia dependent upon excitation without increased blood supply. Dr. B. has found it especially useful in irritable bladder, and the chordee of gleet. We have several times prescribed, ourselves, with benefit in these conditions.

For a careful survey of all the facts Dr. B. gives the following as the *methodus medendi* of the salt in question:

"1st. The bromide of potassium acts by absorption into the blood.

"2d. Its effects are expended upon the nervous centers, or the cerebro-spinal axis.

"3d. Sedation of the heart and circulation, and the various local sedative effects are secondary results of the impression made upon the nervous centers.

"4th. Its physiological effects are not very decided, and are easily modified by any local disturbance.

"5th. Its therapeutical action is still more decidedly influenced by local morbid processes.

"6th. It is indicated where a sedative to the nervous system is required—in insomnia; too great reflex excitability; nervous and spasmodic affections of the larynx and bronchi-sexual excitement, and in an irritable state of the sexual organs.

"7th. It will be effectual in the foregoing conditions, in proportion to the degree in which structural lesions are absent, or in other words, in proportion to the degree in which these morbid states are functional rather than organic."

The bromide, Dr. B. asserts, possesses none of the peculiar alterant property of the iodide. Whilst this fact is true, it is undoubtedly the case that the bromide relieves congestion of certan organs, diminishes their bulk, or, as it may be styled, produces resolutions of an engorgement. Such action, apparently alterative or resolvent, is not really so. It has been exhibited mainly in certain states of the uterus and ovaries—states of hyperæmia dependent upon sexual excitement, or upon the monthly nisus. The apparent resolvent power is, in this ease, due to the sedative impression of the remedy upon the sexual organs and upon the vaso-motor nerves.—*Am. Jour. of Medical Sciences.*

